# Molecular characterization of bovine leukemia virus reveals existence of genotype 4 in Chinese dairy cattle

**DOI:** 10.1186/s12985-019-1207-8

**Published:** 2019-08-27

**Authors:** Yi Yang, Lina Chen, Maoli Dong, Wenjiang Huang, Xiaoli Hao, Yalan Peng, Zaicheng Gong, Aijian Qin, Shaobin Shang, Zhangping Yang

**Affiliations:** 1 Jiangsu Co-innovation Center for Prevention and Control of Important Animal Infectious Diseases and Zoonoses; College of Veterinary Medicine, Yangzhou University, Yangzhou, 225009 Jiangsu China; 2 International Corporation Laboratory of Agriculture and Agricultural Products Safety, Yangzhou University, Yangzhou, 225009 Jiangsu China; 3 Victor Pharmaceutical Company of Limited Liability, Zhenjiang, 212132 Jiangsu China; 4 College of Animal Science and Technology, Yangzhou University, Yangzhou, 225009 Jiangsu China

**Keywords:** Bovine leukemia virus, Molecular characterization, Envelope gene, Genetic diversity

## Abstract

Bovine leukemia virus (BLV) causes enzootic bovine leucosis and is widely spread worldwide, except several European countries, Australia and New Zealand. Although BLV is highly prevalent in China, information about the genetic diversity and evolutionary dynamics of BLV among Chinese dairy herds is still lacking. To determine the genetic variability of BLV, 219 cows from four cities of Ningxia province of China were screened for BLV infection by fluorescence resonance energy transfer (FRET)-PCR and sequencing, 16 selected positive samples were subjected to molecular characterization. Phylogenetic analysis using the neighbor-joining (NJ) method on complete sequences of envelope (*env*) gene of BLV obtained from China and those available in GenBank (representing BLV genotypes 1–10) revealed that those Chinese strains belonged to genotypes 4 and 6. Totally, 23 mutations were identified and 16 of them were determined to be unique mutations among Chinese strains. Alignment of the deduced amino acid sequences demonstrated six mutations in glycoprotein 51 (gp51) and three mutations in glycoprotein 30 (gp30) located in the identified neutralizing domain (ND), CD8^+^ T cell epitope, E-epitope, B-epitope, gp51N12 and cytoplasmic domain of transmembrane protein. This study reported for the first time the BLV genotype 4 in China, and further studies are warranted to compare its immunogenicity and pathogenicity with other BLV genotypes.

**Electronic supplementary material:**

The online version of this article (10.1186/s12985-019-1207-8) contains supplementary material, which is available to authorized users.

## Main text

Bovine leukemia virus (BLV) is the causative agent of enzootic bovine leucosis (EBL), and approximately 30% of infected cattle develop persistent lymphocytosis (PL) while a small percentage of infected cattle die from malignant lymphoma. In recent years, a variety of methods have been applied for BLV genotyping [1–3]. Due to the biological functions, most of studies have primarily focused on the *env* gene. To date, at least 11 genotypes of BLV have been described based on the genetic polymorphism of the *env* gene [4, 5]. Previous studies demonstrated that BLV was widely spread among dairy herds in China, and genotypes 6, 10 and 11 existed in Chinese dairy or yak herds [6, 7].

From November 2018 to January 2019, bovine whole blood samples (*n* = 219) from four cities (Shizuishan, Yinchuan, Wuzhong and Zhongwei) of Ningxia province were submitted to Yangzhou University College of Veterinary Medicine for BLV identification. All samples were freshly collected in ethylenediaminetetraacetic acid (EDTA) blood collection tubes by Center for Animal Disease Control and Prevention of Ningxia province, and delivered on ice with next-day delivery. DNA was extracted from whole blood samples using commercial kit as previously described [8]. The FRET-qPCR targeting BLV *pol* gene (forward primer = 5′-CCTCAATTCCCTTTAAACTAGAACG-3′; reverse primer = 5′-ATGGGCTTTGTAAGAGCATTTGTA-3′; anchor probe = 5′-GACGGGCCAGGCAATAATCCAGT-(6-FAM)-3′; reporter probe = 5′-(LCRed640)-TTCCCGGTACGGAAACCAAATGG-phosphate-3′) was performed following the protocol previous described [9].In total, forty samples were identified to be positive from 219 whole blood samples. Copy numbers of BLV in positive cows ranged from 20 copies/ ml of whole blood to 362,936 copies/ ml of whole blood (mean 19,134 copies/ ml of whole blood and median 35 copies/ ml of whole blood) (Table 1), and those above 130 copies/ ml (*n* = 16) were further identified for genotyping based on the diversity of *env* gene.

**Table 1:** Test result of BLV infection in Ningxia province by FRET-qPCR

City	County	BLV positivity by FRET-qPCR	Copy number range (per ml of whole blood)	Copy number mean (per ml of whole blood)	Copy number median (per ml of whole blood)
Shizuishan	Pingluo	2/17 (11.76%)	1829–6865	4347	4347
	Huinong	3/21 (14.29%)	20–130	57	20
	Dawu	4/14 (28.57%)	20–130	79	83
	Total	9/52 (17.31%)	20–6865	1020	130
Yinchuan	Jinfeng	3/20 (15.00%)	20–362,936	120,992	20
	Yongning	2/17 (11.76%)	35	35	35
	Xingqing	10/20 (50.00%)	20–362,936	37,205	83
	Xixia	0/21 (0.00%)	N/A	N/A	N/A
	Lingwu	6/12 (50.00%)	20–130	41	20
	Total	21/90 (23.33%)	20–362,936	35,016	35
Wuzhong	Litong	4/19 (21.05%)	130–6865	5181	6865
	Yanchi	2/18 (11.11%)	20	20	20
	Qingtongxia	0/21 (0.00%)	N/A	N/A	N/A
	Total	6/58 (10.34%)	20–6865	3461	3498
Zhongwei	Zhongning	4/19 (21.05%)	20	20	20
Total		40/219 (18.26%)	20–362,936	19,134	35

Partial sequence of BLV *env* gene were amplified with an in-house regular PCR [6]. Amplicons were gel purified with the QIAquick Gel Extraction Kit and sequenced with both forward and reverse primers at the GenScript Biotech Corp. (Nanjing, China). Sequence data (PCR products based on the forward and reverse primers) obtained in this study were assembled with DNASTAR Lasergen 15.2 (DNASTAR Inc., Madison, WI) and aligned using CLUSTAL W in MEGA 7.0 (MEGA, Pennsylvania State University, University Park) along with those of BLV strains found on GenBank from around the world. A neighbor-joining (NJ) phylogenetic tree was constructed using the Tamura-Nei model [3, 10] and the robustness of clusters was assessed by bootstrapping 1,000 replicates. Maximum-likelihood (ML) phylogenetic analysis was performed to confirm the results (Additional file 1 Figure S1).

**Fig. 1:**
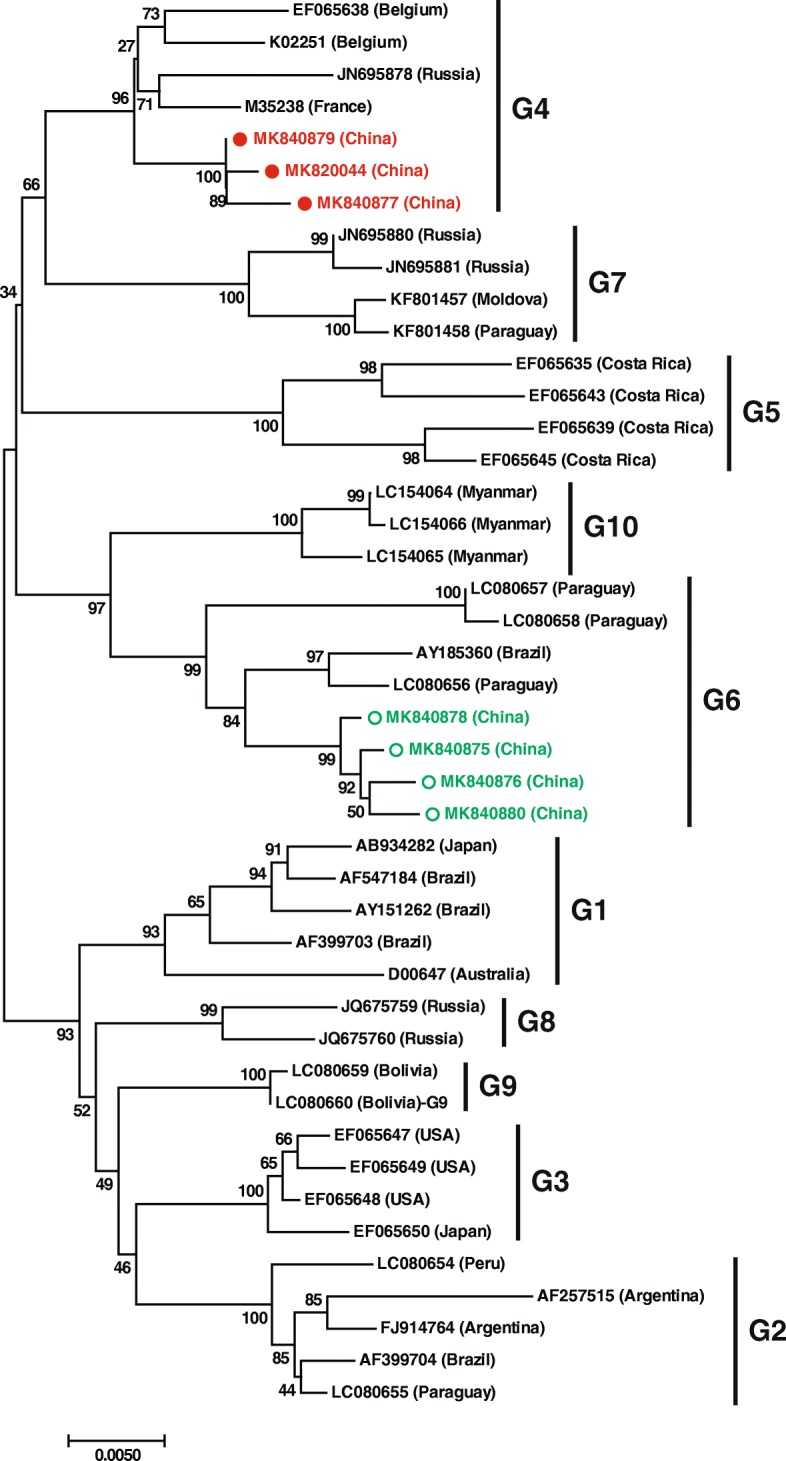
Neighbor-joining phylogenetic tree based on BLV *env* gene complete sequences (1548 bp) from China and around the world**.** Strains identified in our study in China are identified with filled circles (●) for genotype 4 (in red) and open circles (○) for genotype 6 (in green). Genotypes shown on the right are according to Yang et al. [6]. The numbers at the branches show bootstrap support (1000 replicates). The bar at the bottom of the figure denotes distance

Those sequences obtained in this study that were not identical to each other were submitted to GenBank with the GenBank accession numbers: MK820044 and MK840875-MK840880. A neighbor-joining phylogenetic tree based on the *env* gene complete sequences (1,548 bp) of the Chinese strains and 37 reference strains representing BLV genotypes 1 to 10 from 14 countries demonstrated that three Chinese strains (MK820044, MK840877 and MK840879) belonged to genotype 4 and the remaining four (MK840875, MK840876, MK840878 and MK840880) belonged to genotype 6 (Fig. 1).

For those three Chinese strains clustered into BLV genotype 4, the mean distance of the *env* nucleotides and the deduced amino acid (AA) were 0.003 ± 0.001 and 0.008 ± 0.004 between these strains, respectively (Table 2). Compared with the BLV strains obtained from GenBank representing BLV genotypes 1 to 10, the Chinese strains had between 0.014 ± 0.002 and 0.038 ± 0.005 nucleotide distance. Similarly, the Chinese strains had between 0.012 ± 0.003 and 0.037 ± 0.008 deduced AA distance compared with these reference strains (Table 2). The distance of nucleotide and deduced AA indicated that these three Chinese BLV strains were mostly similar to genotype 4 but distinct from genotype 5.

**Table 2:** Nucleotide and amino acid distances (distances ± SE) of BLV *env* gene between Chinese strains and the reference strains

	CHN_G4	CHN_G6	G1	G2	G3	G4	G5	G6	G7	G8	G9	G10
CHN_G4	**0.003±0.001**	0.023±0.006	0.027±0.007	0.029±0.007	0.022±0.007	0.012±0.003	0.037±0.008	0.023±0.006	0.021±0.006	0.024±0.007	0.020±0.006	0.026±0.007
	**0.008±0.004**											
CHN_G6	0.034±0.005	**0.004±0.001**	0.026±0.007	0.029±0.007	0.022±0.007	0.020±0.006	0.037±0.008	0.008±0.003	0.021±0.006	0.019±0.006	0.020±0.006	0.022±0.007
		**0.002±0.002**										
G1	0.032±0.004	0.038±0.005	**0.013±0.002**	0.024±0.005	0.016±0.005	0.026±0.006	0.039±0.008	0.027±0.007	0.024±0.006	0.017±0.005	0.015±0.004	0.032±0.008
			**0.012±0.003**									
G2	0.030±0.004	0.041±0.005	0.028±0.004	**0.011±0.002**	0.013±0.004	0.028±0.006	0.042±0.008	0.028±0.007	0.025±0.006	0.020±0.005	0.017±0.004	0.034±0.007
				**0.020±0.005**								
G3	0.029±0.004	0.037±0.005	0.026±0.004	0.023±0.004	**0.005±0.001**	0.021±0.006	0.035±0.008	0.022±0.007	0.019±0.006	0.013±0.005	0.007±0.004	0.027±0.008
					**0.001±0.001**							
G4	0.014±0.002	0.034±0.005	0.034±0.004	0.033±0.004	0.031±0.005	**0.014±0.002**	0.034±0.007	0.020±0.005	0.019±0.005	0.023±0.006	0.019±0.006	0.024±0.006
						**0.011±0.003**						
G5	0.038±0.005	0.046±0.005	0.042±0.005	0.045±0.005	0.044±0.005	0.038±0.004	**0.020±0.003**	0.036±0.007	0.034±0.007	0.037±0.008	0.034±0.008	0.042±0.008
							**0.021±0.005**					
G6	0.037±0.005	0.020±0.003	0.042±0.005	0.043±0.005	0.038±0.005	0.036±0.005	0.047±0.005	**0.017±0.003**	0.019±0.005	0.019±0.006	0.020±0.006	0.023±0.007
								**0.008±0.003**				
G7	0.029±0.004	0.040±0.006	0.037±0.005	0.037±0.005	0.035±0.005	0.029±0.004	0.044±0.005	0.042±0.005	**0.009±0.002**	0.021±0.006	0.017±0.006	0.026±0.007
									**0.010±0.004**			
G8	0.030±0.004	0.035±0.005	0.026±0.004	0.028±0.004	0.024±0.004	0.032±0.004	0.045±0.005	0.037±0.005	0.037±0.005	**0.011±0.003**	0.011±0.004	0.029±0.008
										**0.005±0.003**		
G9	0.028±0.004	0.034±0.005	0.025±0.004	0.023±0.004	0.019±0.004	0.030±0.004	0.042±0.005	0.038±0.005	0.034±0.005	0.021±0.004	**0.001±0.001**	0.025±0.007
											**0.002±0.002**	
G10	0.031±0.005	0.029±0.005	0.039±0.005	0.040±0.005	0.036±0.005	0.032±0.004	0.044±0.005	0.031±0.004	0.036±0.005	0.037±0.005	0.033±0.005	**0.005±0.002**
												**0.007±0.003**

^a^Left lower diagonal: nucleotide distance among (inter-genotype) BLV genotypes and Chinese strains

^b^Right upper diagonal: amino acid distance among (inter-genotype) BLV genotypes and Chinese strains

^cd^The values in bold along the diagonal are the distance (intra-genotype) of the nucleotides (above) and amino acids (below) between the Chinese strains and those in GenBank

For the remaining four strains clustered into BLV genotype 6, the mean distance of the *env* nucleotides and the deduced AA were 0.004 ± 0.001 and 0.002 ± 0.002, respectively (Table 2). Compared with the BLV strains obtained from GenBank, the Chinese strains had nucleotide distance between 0.020 ± 0.003 and 0.046 ± 0.005. Similarly, the Chinese strains had between 0.008 ± 0.003 and 0.037 ± 0.008 deduced AA distance compared with those reference strains (Table 2). The distance of nucleotide and deduced AA indicated that the three Chinese BLV strains were mostly similar to genotype 6 but distinct from genotype 5.

**Fig. 2:**
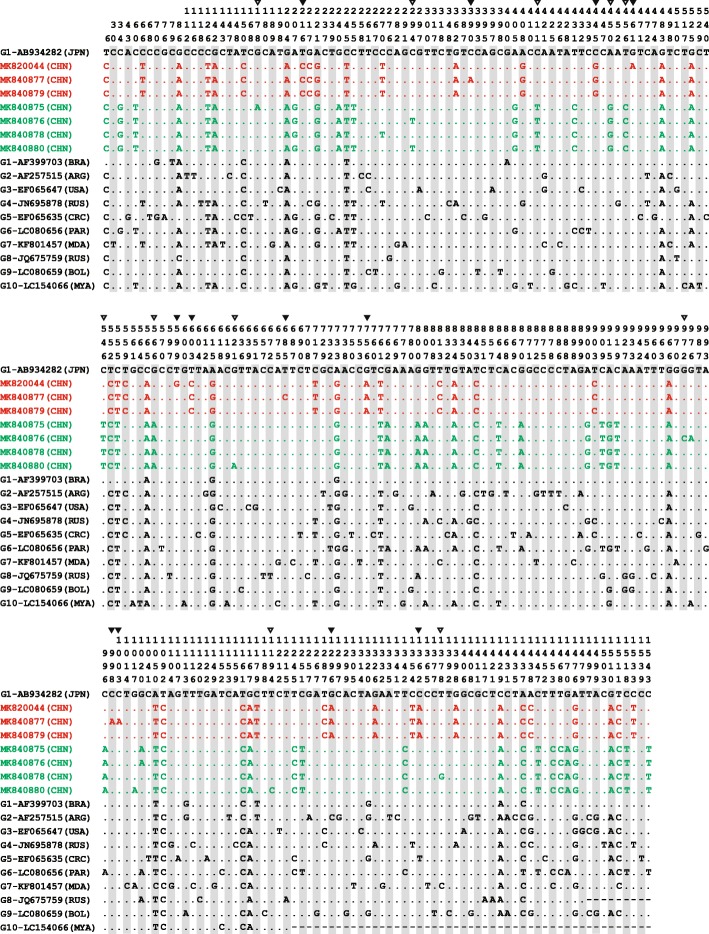
Alignment of full-length of BLV *env* gene nucleotide sequences (1548 bp) between sequences obtained in this study together with 10 reference sequences obtained in the GenBank database. Strains identified in this study are in red (cluster into genotype 4) and green (cluster into genotype 6). Numbers above the sequences are nucleotide number indicated by the *env* gene of AB934282. The countries of the strains are marked with abbreviations in parentheses to the right of the GenBank accession numbers. Dots indicate nucleotides identical to the reference sequences. The mark above the square frames indicate mutations for our isolates of BLV genotype 4 (▼) and genotype 6 (▽). The BLV reference strains from GenBank have accession numbers AF933703 (G1), AF257515 (G2), EF065647 (G3), JN695878 (G4), EF065635 (G5), LC080656 (G6), KF801457 (G7), JQ675759 (G8), LC080659 (G9), and LC154066 (G10). The seven Chinese strains from this study have the accession numbers MK820044 and MK840875-MK840880. JPN = Japan; CHN = China; BRA = Brazil; ARG = Argentina; USA = United States of America; RUS = Russia; CRC = Costa Rica; PAR = Paraguay; MDA = Moldova; BOL = Bolivia; MYA = Myanmar

Although 10 genotypes of BLV have been discovered around the world, there is little information on genetic diversity of BLV among Chinese dairy herds [11], until BLV genotype 6 was firstly identified in Yancheng, Shanghai, Yangzhou, Bengbu and Tianjin in 2019 [6]. The present study revealed the existence of BLV genotype 4 in China for the first time. When compared with the reference sequences representing all 10 BLV genotypes deposited in GenBank, we found that our Chinese isolates had a total of 23 mutations in complete *env* gene. Among them, fourteen were synonymous mutations (T216C, C294T, C390A, C411T, A450G, T456C, C546T, T603C, G621A, G756A, G972C, T1194C, G1296A and C1362A) and the remaining nine were nonsynonymous mutations (G178A, C445G, G461A, G566A, T599G, T685C, C998A, C1003A and T1378G). Interestingly, the Chinese strains of BLV genotype 4 and genotype 6 have 12 and 11 mutations, respectively on the *env* gene that was not shared between the two groups (Fig. 2). The distance between BLV genotype 4 and 6 was 0.036 ± 0.005 (nucleotide) and 0.020 ± 0.005 (AA). When compared with the 10 reference sequences and all Chinese sequences available in the GenBank database, 16 of the mutations (G178A, T216C, C390A, C445G, G461A, T599G, T603C, T685C, G756A, G972C, C998A, C1003A, T1194C, G1296A, C1362A and T1378G) were identified as unique mutations (Additional file 2 Figure S2-A and S2-B).

**Fig. 3:**
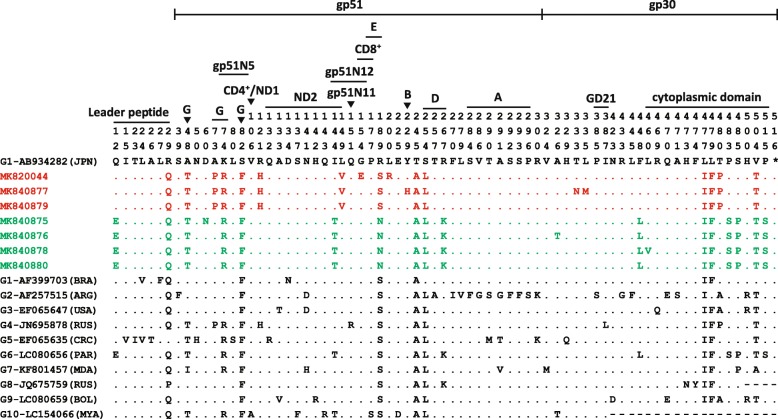
Alignment of full-length of BLV *env* gene amino acid sequences (515 AA) between Chinese and reference strains. Chinese strains identified in this study are in red (cluster into genotype 4) and green (cluster into genotype 6). Numbers above the sequences are AA residue number indicated by the *env* gene of AB934282. The countries of the strains are marked with abbreviations in parentheses to the right of the GenBank accession numbers. Dots indicate amino acid identical to the reference sequence. Labeled lines and ▼ indicate the position of identified glycoproteins, conformational epitope (G), linear epitopes (A, B, D and E), CD4^+^ and CD8^+^ T cell epitopes, gp51N5, gp51N11 and gp51N12, peptides, neutralization domains (ND1 and ND2) and cytoplasmic domain of transmembrane protein. The BLV reference strains from GenBank have accession numbers AF933703 (G1), AF257515 (G2), EF065647 (G3), JN695878 (G4), EF065635 (G5), LC080656 (G6), KF801457 (G7), JQ675759 (G8), LC080659 (G9), and LC154066 (G10). The seven Chinese strains from this study have the accession numbers MK820044 and MK840875-MK840880. JPN = Japan; CHN = China; BRA = Brazil; ARG = Argentina; USA = United States of America; RUS = Russia; CRC = Costa Rica; PAR = Paraguay; MDA = Moldova; BOL = Bolivia; MYA = Myanmar

Among all the nonsynonymous mutations, seven of them (G178A, G461A, T599G, T685C, C998A, C1003A and T1378G) were observed in single isolate and the remaining two (C445G and G566A) were existed in three or four isolates. Alignment of deduced amino acid sequences demonstrated that six mutations (D60N, L149 V, G154E, R189N, L200R and Y229H) were in glycoprotein 51 (gp51) and three mutations (T333 N, L335 M and L460 V) were in glycoprotein 30 (gp30), distributing respectively in the neutralizing domain 2, CD8^+^ T cell epitope, E-epitope, B-epitope, gp51N12 and cytoplasmic domain of the transmembrane protein [12–14] (Fig. 3).

This study investigated the prevalence and genetic variability of BLV and identified the BLV genotype 4 in China for the first time. Together with our previous study [6] and studies conducted by Wang and Yu in 2018 and 2019 [5, 7], BLV genotypes 1, 4, 6 and 10 were present in dairy cattle or yaks in China. BLV genotype 4 is the second most common genotype prevalent worldwide and was identified in Mongolia in 2016 [2]. The cow trade between China and Mongolia might contributed to the spread of BLV between the two countries. This study will help us to better understand the genetic diversity of BLV in China. However, further studies are needed to define the immunogenicity and pathogenicity between different genotypes of BLV.

## Additional files

Additional file 1: **Figure S1.** Maximum-likelihood phylogenetic tree based on BLV env gene complete sequences (1548 bp) from China and around the world. Strains identified in our study in China are identified with filled circles (●) for genotype 4 (in red) and open circles (○) for genotype 6 (in green). Genotypes shown on the right are according to Yang et al. [6]. The numbers at the branches show bootstrap support (1000 replicates). The bar at the bottom of the figure denotes distance. (AI 1649 kb)

Additional file 2 **Figure S2.** Alignment of full-length of BLV *env* gene nucleotide sequences (S2-A: 6–769 bp; S2-B: 777–1543 bp) between sequences obtained in this study together with 10 reference sequences and all Chinese sequences available in the GenBank database. Strains identified in this study are in red (cluster into genotype 4) and green (cluster into genotype 6). Numbers above the sequences are nucleotide number indicated by the env gene of AB934282. The countries of the strains are marked with abbreviations in parentheses to the right of the GenBank accession numbers. Dots indicate nucleotides identical to the reference sequences. The mark above the square frames indicate unique mutations for our isolates of BLV genotype 4 (▼) and genotype 6 (▽). The BLV reference strains from GenBank have accession numbers AF933703 (G1), AF257515 (G2), EF065647 (G3), JN695878 (G4), EF065635 (G5), LC080656 (G6), KF801457 (G7), JQ675759 (G8), LC080659 (G9), and LC154066 (G10). The Chinese sequences available in the GenBank database have the accession numbers: MH040198-MH040203, MH040205, MH040207-MH040209, MF574053-MF574068. The seven Chinese strains from this study have the accession numbers MK820044 and MK840875-MK840880. JPN = Japan; CHN = China; BRA = Brazil; ARG = Argentina; USA = United States of America; RUS = Russia; CRC = Costa Rica; PAR = Paraguay; MDA = Moldova; BOL = Bolivia; MYA = Myanmar. (ZIP 5157 kb)

## Data Availability

The sequences of full-length envelope gene generated in this study have been deposited in GenBank under the accession numbers MK820044 and MK840875-MK840880.

## Abbreviations

AA: amino acid
ARG: Argentina
BLV: Bovine leukemia virus
BOL: Bolivia
BRA: Brazil
CHN: China
CRC: Costa Rica
EBL: Enzootic bovine leucosis
EDTA: Ethylenediaminetetraacetic acid
Env: envelope
FRET: Fluorescence resonance energy transfer
gp30: glycoprotein 30
gp51: glycoprotein 51
JPN: Japan
MDA: Moldova
ML: Maximum likelihood
MYA: Myanmar
ND: Neutralizing domain
NJ: Neighbor-joining
PAR: Paraguay
PL: Persistent lymphocytosis
RUS: Russia
USA: United States of America
